# Simultaneous detection of neurotransmitters and Cu^2+^ using double-bore carbon fiber microelectrodes *via* fast-scan cyclic voltammetry†

**DOI:** 10.1039/d3ra06218j

**Published:** 2023-11-20

**Authors:** Noel Manring, Miriam Strini, Jessica L. Smeltz, Pavithra Pathirathna

**Affiliations:** a Department of Chemistry and Chemical Engineering, Florida Institute of Technology Melbourne FL USA ppathirathna@fit.edu

## Abstract

There is a great demand to broaden our understanding of the multifactorial complex etiology of neurodegenerative diseases to aid the development of more efficient therapeutics and slow down the progression of neuronal cell death. The role of co-transmission and the effect of environmental factors on such diseases have yet to be explored adequately, mainly due to the lack of a proper analytical tool that can perform simultaneous multi-analyte detection in real time with excellent analytical parameters. In this study, we report a simple fabrication protocol of a double-bore carbon-fiber microelectrode (CFM) capable of performing rapid simultaneous detection of neurotransmitters and Cu^2+^*via* fast-scan cyclic voltammetry (FSCV) in Tris buffer. After imaging our CFMs *via* optical microscopy and scanning electron microscopy to ensure the intact nature of the two electrodes in our electrode composite, we performed a detailed analysis of the performance characteristics of our double-bore CFM in five different analyte mixtures, Cu^2+^-5HT, Cu^2+^-DA, Cu^2+^-AA, 5-HT-DA, and 5-HT-AA in Tris buffer, by applying different analyte-specific FSCV waveforms simultaneously. Calibration curves for each analyte in each mixture were plotted while extracting the analytical parameters such as the limit of detection (LOD), linear range, and sensitivity. We also carried out a control experiment series for the same mixtures with single-bore CFMs by applying one waveform at a time to compare the capabilities of our double-bore CFMs. Interestingly, except for the Cu^2+^-DA solution, all other combinations showed improved LOD, linear ranges, and sensitivity when detecting simultaneously with double-bore CFMs compared to single-bore CFMs, an excellent finding for developing this sensor for future *in vivo* applications.

## Abstract 2

There is a great demand to develop an analytical tool capable of simultaneous detection of multi neurotransmitters and other environmental factors to broaden our understanding of the multifactorial complex etiology of neurodegenerative diseases.

## Graphical abstract

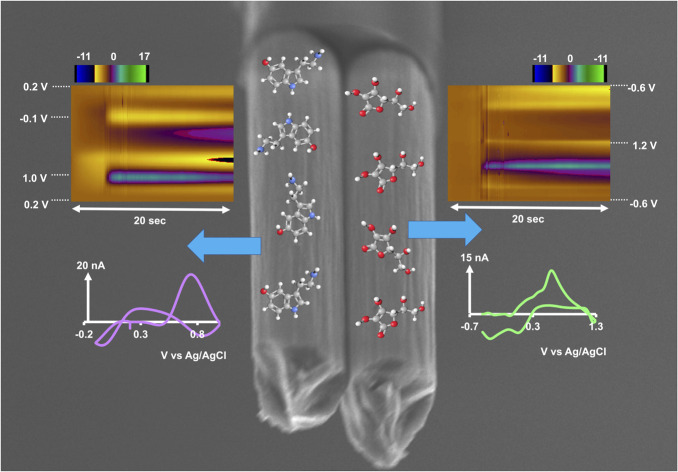

## Introduction

The prevalence of neurological disorders is on the rise globally despite the efforts taken by scientists, the pharmaceutical industry, and medical professionals to develop medicines that slow down the progression of the disease. Despite the extensive research performed on understanding the relationship between aging and the onset of neurological disorders, it is recognized that the etiology of such illnesses is multifactorial, and more attention is required to explore the effect of environmental factors such as heavy metals.^1–5^ Similarly, little experimental information is available on the regulation and functional effects of co-transmission, a ubiquitous feature of the nervous system that presumably plays a vital role in controlling the death of neurons. Although it has been nearly 45 years since the concept of “co-transmission” was introduced,^6–10^ most early studies have been performed with lower vertebrate model systems. More recent studies with optogenetics^11,12^ reveal more accurate information related to higher vertebrate systems;^13,14^ however, there are limitations associated with these measurements. For example, optogenetic activation of neurons may not resemble physiological conditions. Therefore, there is still a critical gap in the knowledge base related to accurate co-transmission information. The major limitation to revealing such vital information is the lack of a proper tool to perform simultaneous *in vivo*, real-time measurements of neurotransmitters and other substances that play a critical role in neurodegenerative diseases.

Among several detection strategies to monitor neurotransmitters in the brain, many researchers have extensively used fast-scan cyclic voltammetry (FSCV) due to its excellent temporal resolution.^15,16^ Carbon fiber microelectrodes (CFMs) are frequently used with FSCV due to minimal cell damage caused by ultra-small electrodes (5–7 μm) and their superior biocompatibility compared to other electrode materials. However, most studies are limited to single-analyte and single-site^17–19^ measurements, and several attempts have been taken to improve sensitivity, selectivity and decrease fouling of electrodes.^20–22^ In contrast to using CFMs and FSCV for simultaneous measurements of neurotransmitters, a few studies have been conducted with other electrode materials and slow scan cyclic voltammetry. Wang and colleagues fabricated a modified electrode with intercalated carbon nanotubes on a graphite surface and used it to detect dopamine (DA) and serotonin (5-HT) in the presence of ascorbic acid (AA) in a rabbit's brain homogenate using differential pulse voltammetry (DPV) at 50 mV s^−1^.^23^ Similarly, Kachoosangi and Compton demonstrated that unmodified edge plane pyrolytic graphite electrodes could be used to simultaneously detect DA, 5-HT with AA in phosphate buffer using DPV.^24^ Although these studies report well-resolved unique cyclic voltammograms and peaks for each neurotransmitter, these methods lack the temporal resolution needed for *in vivo*, real-time detection.

Among a few of the attempts of co-detection of neurotransmitters, Swamy and Venton used carbon-nanotube-modified single CFMs to detect DA and 5-HT in mice.^25^ However, their analysis was based on identifying reduction peaks of DA and 5-HT at different time scales. Recently, Castagnola *et al.* introduced a new glassy carbon array microelectrode that can simultaneously perform *in vivo* FSCV measurements to detect DA and 5-HT.^26^ However, their electrodes were designed through a highly complex microfabrication procedure that involves six lengthy steps. Moreover, the Amemiya group fabricated a double-bore CFM with a nanometer-wide gap to study the nanogap-based kinetics of Ru(NH_3_)_6_^3+/2+^, DA, and AA. Although the Amemiya group was able to maintain the nanogap between two electrodes with a greater probability, they were unable to cycle the potential at both electrodes. They applied a constant potential to one of the electrodes and performed amperometric measurements while they cycled the potential at the other electrode, performing cyclic voltammetric measurements at 100 V s^−1^. Amperometry is not ideal for quantifying neurotransmitters in the brain due to poor selectivity in the complex matrix. Moreover, the scan rate they used in their study wasn't ideal for fast, real-time *in vivo* measurements.^27^

In this study, for the first time, we report the fabrication of a simple, manually cut double-bore CFM capable of ultrafast simultaneous detection of neurotransmitters and Cu^2+^ using FSCV with a temporal resolution of 100 m s^−1^. Using the oscilloscope images, we first tested our double-bore CFMs' ability to withstand two different waveforms cycling at faster scan rates *via* FSCV. We then performed FSCV measurements using our double-bore CFMs with five different analyte combinations: Cu^2+^ with DA, Cu^2+^ with 5-HT, Cu^2+^ with AA, 5-HT with DA, and 5-HT with AA. We constructed calibration curves of each combination of the analytes at varying concentrations in Tris buffer. We sought to understand how the simultaneous detection of our target analytes differed from the detection of one with the presence of the other in the Tris buffer matrix. To investigate this, we conducted a series of interference tests using 50 μm single-bore CFMs with the same combinations of our target analytes and constructed calibration curves. Our sensor showcases the advantage of using a double-bore CFM, by obtaining a lower limit of detections (LODs) and higher sensitivities compared to a single-bore CFM. We also imaged our electrodes with SEM to identify a ∼640 nm sized gap between the two CFMs. To the best of our knowledge, this is the first report of using a double-bore CFM to detect Cu^2+^ and neurotransmitters using FSCV simultaneously. The high sensitivity and stability of our sensor in Tris buffer that mimics artificial cerebellum fluid show the great potential for using these electrodes in future *in vivo* studies.

## Materials and methods

### Chemicals

Unless otherwise specified, chemicals were purchased from Sigma-Aldrich (St. Louis, MO, USA). Dopamine hydrochloride (Alfa Aesar, Tewksbury, MA, USA) was used to prepare DA solutions. Cupric sulfate (Fisher Scientific, Hampton, NH, USA) was used as the Cu^2+^ source. Serotonin Hydrochloride (TCI Chemicals Montgomeryville, PA, USA) was used to prepare 5-HT solutions. l-Ascorbic acid (Fisher Scientific, Hampton, NH, USA) was used to prepare AA solutions. All analyte solutions were prepared in Tris buffer composed of tris hydrochloride (15 mM), NaCl (140 mM), KCl (3.25 mM), CaCl_2_ (1.2 mM), NaH_2_PO_4_ (1.25 mM), MgCl_2_ (1.2 mM), and Na_2_SO_4_ (2.0 mM) at pH 7.4.

### Fabrication of double-bore carbon fiber microelectrodes

Two individual carbon fibers (diameter: 7 μm, Goodfellow, Pittsburgh, PA, USA) were inserted into two bores on the diagonal in a four-bore borosilicate glass capillary (bore diameter of 0.015′′ and outer diameter of 0.062′′, Friedrich and Dimmock, Millville, NJ) utilizing electrostatic forces between a wire and carbon fibers. Unlike previously reported methods,^27–29^ no special vacuum system was used in this step. The fiber-filled capillaries were pulled under gravity using a vertical puller, PE-100 (Narishige Group, Setagaya-Ku, Tokyo, Japan), producing two separate carbon-glass seals. The pulled CFMs were then trimmed manually to 40–50 μm under an optical microscope (Unitron Examet-5 series, Commack, NY, USA), a cheap, easy method compared to previously reported methods.^27^

### Fabrication of single-bore CFMs

A carbon fiber (diameter: 7 μm, Goodfellow, Pittsburgh, PA, USA) was inserted into a glass capillary (outer diameter of 1.0 mm and inner diameter of 0.58 mm, Sutter Instruments, CA, USA) as previously reported^17,18,30,31^ except not using any vacuum pumps to insert the fiber but to utilize electrostatic forces between the fiber and the glass capillary as mentioned above.

### Electrochemical measurements

All electrochemical measurements were performed in a two-electrode system with CFMs as working electrodes and in-house built Ag/AgCl electrode as the reference electrode. FSCV experiments (data collection, analysis, and background subtraction) were performed with the Quad-UEI system (Electronics Design Facility, University of North Carolina, Chapel Hill, NC, USA). Each electrochemical experiment was conducted with at least 4 CFMs in triplicate (at least 12 runs in total). All values were reported with the average of all replicate measurements and the standard error of the mean.

### Imaging

Double-bore CFMs were imaged with an optical microscope (Unitron Examet-5 series, Commack, NY, USA) and a scanning electron microscope (SEM) (JSM-6380/LV, Jeol Ltd. Tokyo, Japan) located in the High-Resolution Microscopy and Advanced Imaging Center at the Florida Institute of Technology. Images were taken at 7500 and 1700 magnification at 30 kV.

## Results and discussion

### Fabrication of double-bore CFMs

Uncovering the interactions of two or more neurotransmitters in real time is extremely helpful in broadening the current understanding of brain diseases and investigating new, efficient therapeutics. Although several configurations of multi-electrodes have been reported,^23–27^ simultaneous detection of multiple neurotransmitters with other analytes in real-time hasn't been successfully achieved yet due to the incompatibility of electrode configuration and lack of analytical capabilities with existing systems. Among various geometries of electrodes, cylindrical-shaped single CFMs have been successfully used as implantable electrodes for *in vivo* experiments with mice models owing to their smaller size that enhances mass transport through hemispherical diffusion with minimal cell damage.^32,33^ Interestingly, the Amemiya group reported a cylindrical double-bore CFM fabricated using focused ion beam milling (FIB) capable of maintaining a stable gap with a success rate of 62.5%.^27^ Although their milling was 100% successful *via* FIB, it's an expensive and tedious method to fabricate CFMs. Furthermore, they were not able to cycle the potential across two electrodes simultaneously, thus, limiting its application towards real-time co-detection of multi-neurotransmitters with excellent selectivity. In this study, we modified the fabrication protocol reported by the Amemiya group to eliminate expensive FIB milling for electrode trimming while maintaining a stable gap. Although our trimming rate is ∼90%, we were always able to maintain an intact gap with a 100% success rate after completing the fabrication process.

We used a four-bore borosilicate glass capillary to fabricate our two-bore CFMs by inserting two carbon fibers into two bores along the diagonal, leaving the other two bores empty. The rationale behind using a four-bore glass capillary instead of a two-bore glass capillary was to maintain a small yet more stable gap between the two electrodes to avoid cross-talking while applying two different waveforms at faster scan rates. We performed the same experiments first with double-bore capillaries. Although we were able to maintain a stable gap between two fibers when the same waveform was applied, the gap was closed upon using two different waveforms with different polarities (data not shown here).

**Fig. 1:**
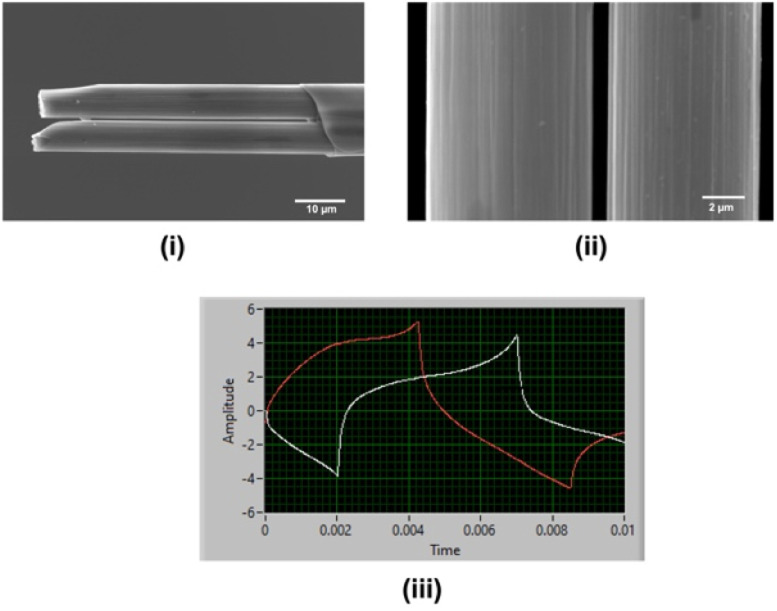
SEM images of a double-bore CFM after manually trimming under optical microscope (i), the gap between two carbon fibers (ii) and the oscilloscope image when two separate waveforms applied to double-bore CFMs (iii). Here, the red line corresponds to the DA waveform (−0.6 V to +1.2 V at 400 V s^−1^) and the white line to the Cu^2+^ waveform (−0.7 V to +1.1 V at 400 V s^−1^).

Carbon fibers were inserted using the electrostatic forces between the glass and fibers, a different yet simpler and easier approach than previously reported methods.^27–29^ This method eliminates using any vacuum system to insert carbon fibers. A filled glass capillary with carbon fibers was then pulled using a heat-puller under gravitational force, and the exposed carbon fibers were trimmed to ∼40–50 μm manually. Pulled glass capillaries were firmly mounted on two strips of blue tack to prevent possible damage to glass capillaries while manually cutting. Although the cutting edges (Fig. 1(i)) were not as sharp and less precise compared to the FIB-milled CFMs,^27^ this approach was faster, simpler, and inexpensive. Although the gap between the two fibers was apparent under an optical microscope (Fig. S1†), it was further confirmed by imaging *via* SEM. The particle seen between the two fibers is probably a dust particle trapped when mounting the CFM on the SEM stage. As seen in Fig. 1(ii), the gap between the two fibers was ∼640 nm when SEM images were taken in the air.

Fig. 1(iii) depicts the oscilloscope image taken after placing the double-bore CFM in Tris buffer and simultaneously applying Cu^2+^-waveform (white) at 400 V s^−1^ to one electrode and DA-waveform (red) at 400 V s^−1^ to the other with 10 Hz frequency. As seen in this oscilloscope image, there is no overlap between the signals, and no electrical noise is seen on either of the signals, confirming that electrodes were able to maintain a gap even in the solution upon cycling two different potential windows simultaneously. This is an exciting finding, as it is crucial to keep these electrodes intact when performing co-detection of multiple analytes. To the best of our knowledge, this is the first time reporting a successful double-bore CFM capable of applying two different waveforms using FSCV with no electrical interference at 100 ms temporal resolution.

### Simultaneous detection of neurotransmitters and toxic metals

**Fig. 2:**
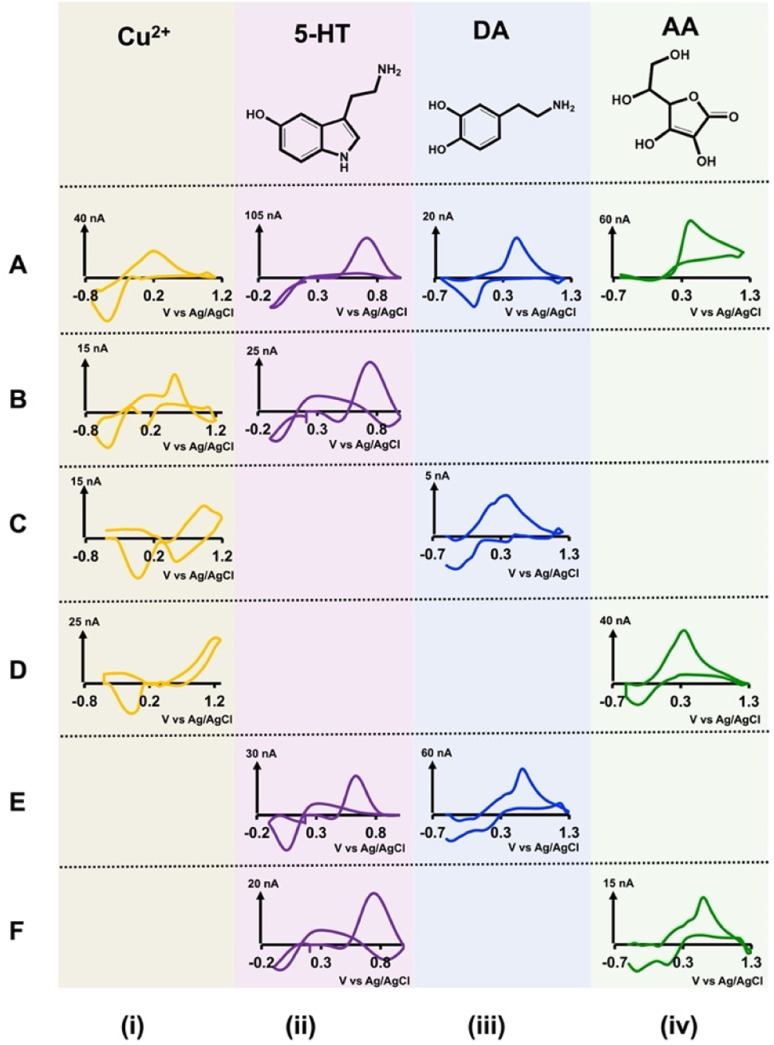
Top row (A) shows representative CVs obtained for each analyte solution in Tris buffer with single-bore CFMs using analyte-specific waveforms. The concentration of each analyte for these representative CVs are 5 μM Cu^2+^ (A(i)), 1 μM 5-HT (A(ii)), 0.5 μM DA (A(iii)), and 50 μM AA (A(iv)). Rows (B)–(F) show representative CVs obtained for each analyte mixture with double-bore CFMs when applying two different analyte-specific waveforms simultaneously. These analyte mixtures are (B) 2 μM Cu^2+^ (i) and 0.25 μM 5-HT (ii), (C) 5 μM Cu^2+^ (i) and 0.5 μM DA (iii), (D) 5 μM Cu^2+^ (i) and 100 μM AA (iv), (E) co-detection of 1 μM 5-HT (ii) and 1 μM DA (iii), and (F) 0.25 μM 5-HT (ii) and 5 μM AA (iv).

After confirming that our electrodes could stay intact in the solution, we performed simultaneous FSCV measurements of DA, 5-HT, AA, and Cu^2+^ in Tris buffer. DA, 5-HT, and AA were chosen since these are commonly found in the brain and are linked with many neurological disorders,^34,35^ and Cu^2+^ was selected as a model toxic metal ion that has been studied extensively *via* FSCV^30,31,36,37^ and is linked to some neurodegenerative diseases such as Alzheimer's.^38–40^ Here, we prepared solutions of Cu^2+^-DA, Cu^2+^-5-HT, Cu^2+^-AA, 5-HT-DA, and 5-HT-AA in Tris buffer with varying concentration ratios and performed FSCV measurements by applying analyte-specific waveform for each electrode. We constructed calibration curves (Fig. S2 and S3†) to determine each solution mixture's LOD, linear range, and sensitivity for each analyte. Fig. 2 depicts representative CVs from each solution combination (rows (B) through (F)) and Table 1(A) represents the summary of analytical parameters for each of those solution mixtures. Similarly, the representative color plots for each combination are presented in Fig. S4 and S5.† Because the analytical performance of each combination is varied, each CV pair in Fig. 1 was chosen from different concentration combinations.

LOD, linear ranges, and sensitives for co-detection with double-bore CFMs (A) and with single-bore CFMs in different analyte mixtures (B)

**Table:** 

(A) Analytical parameters for double-bore CFMs							
Analyte 1	Analyte 2	LOD (μM)		Linear range (μM)		Sensitivity (nA μM^−1^)	
		Analyte 1	Analyte 2	Analyte 1	Analyte 2	Analyte 1	Analyte 2
Cu^2+^	5-HT	0.25	0.025	0.25–2	0.025–0.5	7.21	53.09
Cu^2+^	DA	1	0.1	1–10	0.1–1	1.58	8.87
Cu^2+^	AA	0.25	2	0.25–5	2–50	6.50	0.54
5-HT	DA	0.05	0.05	0.05–1	0.05–1	35.35	36.59
5-HT	AA	0.05	1	0.05–1	1–10	32.33	2.64

**Table:** 

(B) Analytical parameters for single-bore CFMs							
Analyte 1	Analyte 2	LOD (μM)		Linear range (μM)		Sensitivity (nA μM^−1^)	
		Analyte 1	Analyte 2	Analyte 1	Analyte 2	Analyte 1	Analyte 2
Cu^2+^	5-HT	0.5	0.05	0.5–5	0.05–1	3.06	25.62
Cu^2+^	DA	1	0.1	1–10	0.1–1	1.58	8.87
Cu^2+^	PA	1	10	1–5	10–100	3.48	0.31
5-HT	DA	0.1	0.1	0.1–1	0.1–1	15.11	14.83
5-HT	AA	01	10	01–1	10–250	16.49	0.36

We also obtained CVs for each analyte (top row (A) in Fig. 2) in Tris buffer with single-bore CFMs (length ∼50 μm) *via* FSCV as a reference to understand our double-bore CVs. Researchers have been using the characteristic shapes of these CVs for qualitative analysis and analyte-specific reduction or oxidation peaks for quantitative analysis. For example, the reduction peak of Cu^2+^^30^ and the oxidation peak of 5-HT,^41^ DA,^27^ and AA^27^ are used for identification and analysis.

When analysing different CVs of Cu^2+^ (Fig. 2B(i)–D(i)), although the reduction peak is visible, there are some notable differences among different solution mixtures. In the presence of 5-HT, the reduction peak of Cu^2+^ CV appears ∼ −0.45 V (Fig. 2B(i)), and it's nearly identical to that of a characteristic Cu^2+^ CV (Fig. 2A(i)).^30^ However, instead of one oxidation peak, two oxidation peaks can be seen (Fig. 2A(ii)). We hypothesize this is because of the combination of oxidation peaks of Cu^2+^ and 5-HT. When Cu^2+^ CVs are taken in the presence of DA, two reduction peaks appear with a distorted oxidation peak (Fig. 2C(i)). While the reduction of Cu^2+^ to Cu^0^ is forced by applying the Cu^2+^-specific waveform,^31^ a complex between Cu^2+^-DA may undergo another reduction, resulting in an extra peak in the presence of another waveform on the adjacent electrode. Furthermore, the magnitudes of both of these reduction peaks change with the concentration (data not shown); thus, it's probable that a complex is formed between Cu^2+^ and DA. In the presence of AA, both the reduction and oxidation peaks of Cu^2+^ appear at more positive potentials. Although there are some notable differences in the oxidation peaks of these Cu^2+^ CVs, the characteristic reduction peak is still preserved with minimum potential shift, thus making the identification of Cu^2+^ possible in these solutions mixtures.

Interestingly, all 5-HT CVs have the characteristic shape and the oxidation peak in all solution mixtures, with a slight exception in 5-HT-DA solution (Fig. 2B(ii), E(ii), and F(ii)). Although the oxidation peaks in 5-HT and DA CVs appear ∼0.66–0.68 V, the maximum oxidation currents on each CV are more or less similar to the expected currents for 1 μM for each analyte, confirming that there are no overlaps on these peaks. Furthermore, it has been found that DA-specific waveform was not sensitive to detecting 5-HT.^42^ The more prominent reduction peak on the 5-HT CV is most likely coming from the DA-reduction.

Both DA CVs obtained with Cu^2+^ and 5-HT (Fig. 2C(iii) and E(iii)) have DA's specific oxidation peaks; thus, identifying DA in these mixtures is possible even with distorted reduction peaks. Similarly, the characteristic AA oxidation peak is seen on both AA CVs in two solutions with Cu^2+^ (Fig. 2D(iv)) and 5-HT (Fig. 2F(iv)). Dehydroascorbic acid, the oxidative product of AA, is short-lived and rapidly hydrolyzes to redox inactive;^43^ species, thus, only a prominent oxidation peak is seen at faster scan rates. However, an interesting reduction peak around ∼0.27 V is seen with our double-bore CFM, presumably due to the stabilizing of dehydroascorbic acid by Cu^2+^/Cu^0^ system in the presence of a secondary electric field on the other electrode. This enables the reduction peak to be captured by our double-bore CFM. Furthermore, the magnitude of this reduction peak is concentration-dependent (data not shown). DA-AA analyte mixture was not tested as a separate combination since the same waveform is used for both these analytes, and their oxidation peaks appear in the same potential range.

### Detection of analyte mixtures with single-bore CFMs

Although our double-bore CFMs resulted in unique, distinguishable CVs for each analyte in different solution mixtures, we further confirmed any possible significant overlapping in those analyte CVs by analyzing each combination with single-bore CFMs. We prepared a series of concentrations for each solution mixture (Cu^2+^-5-HT, Cu^2+^-DA, Cu^2+^-AA, 5-HT-DA, and 5-HT-AA) in Tris buffer and analyzed these with single-bore CFMs by applying one waveform at a time. We trimmed our single-bore CFMs to ∼40–50 μm to maintain the same length as our double-bore CFMs. Moreover, we constructed calibration curves (Fig. S6 and S7†) with each solution to define analytical parameters (Table 1B).

**Fig. 3:**
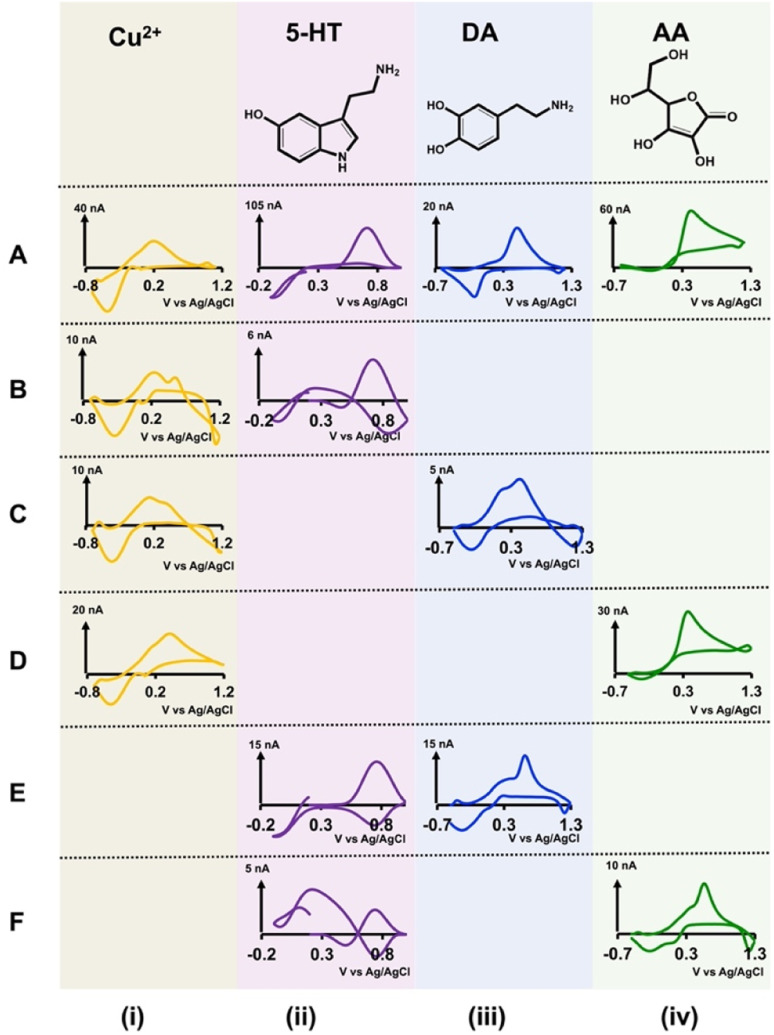
Top row (A) shows representative CVs obtained for each analyte solution in Tris buffer with single-bore CFMs using analyte-specific waveforms. The concentration of each analyte for these representative CVs are 5 μM Cu^2+^ (A(i)), 1 μM 5-HT (A(ii)), 0.5 μM DA (A(iii)), and 50 μM AA (A(iv)). Rows (B)–(F) show representative CVs obtained for each analyte mixture with single-bore CFMs when applying each analyte-specific waveform at a time. These analyte mixtures are (B) 2 μM Cu^2+^ (i) and 0.25 μM 5-HT (ii), (C) 5 μM Cu^2+^ (i) and 0.5 μM DA (iii), (D) 5 μM Cu^2+^ (i) and 100 μM AA (iv), (E) detection of 1 μM 5-HT (ii) and 1 μM DA (iii), and (F) 0.25 μM 5-HT (ii) and 5 μM AA (iv).

In Fig. 3, the same traditional CVs for each analyte were included in the top row, A, for comparison purposes. As seen in the first column (Fig. 3B(i)–D(i)), the characteristic reduction peak of Cu^2+^ is seen in the presence of the other three analytes. Interestingly, the second reduction peak that appeared with double-bore CFM in Cu^2+^-DA solution (Fig. 2C(i)) is absent when detecting with a single-bore CFM, and this confirms our hypothesis regarding the reduction of a possible secondary Cu^2+^-DA complex in the presence of a second electric field. Unlike with double-bore CFMs, 5-HT CVs in analyte mixtures (Fig. 3B(ii), E(ii), and F(ii)) are distorted significantly from that of an ideal 5-HT CV shown in the top row. The CVs obtained for DA (Fig. 3C(iii) and E(iii)) and AA (Fig. 3D(iv) and F(iv)) in other solution mixtures have the characteristic oxidation peaks for DA and AA with small shifts in the potentials and slight distortions of reduction peaks. Moreover, the reduction peak that appeared on AA CV in the presence of Cu^2+^ with double-bore CFM is absent with single CFM, confirming the expected loss of the unstable, oxidative product of AA, dehydroascorbic acid.^43^ Interestingly, this comparison study shows that the CVs obtained with double-bore CFMs are superior both in shape and magnitude to those obtained with single-bore CFMs, except for the Cu^2+^-DA mixture. This is an excellent feature when developing this sensor for *in vivo* measurements.

### Comparison of analytical parameters

**Fig. 4:**
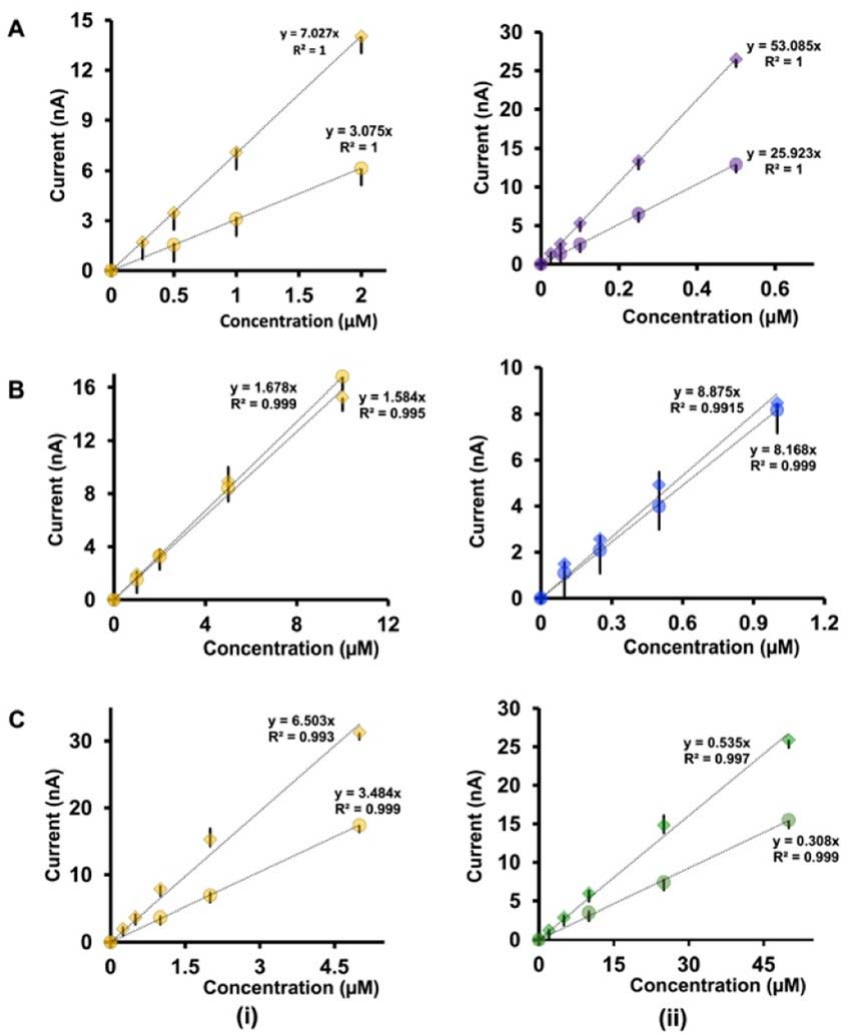
Comparison of the linear ranges of calibration curves obtained with double-bore CFMs by applying two waveforms simultaneously (diamonds) and with single-bore CFMs by applying one waveform at a time (circles) for (A) Cu^2+^-5-HT (B) Cu^2+^-DA, and (C) Cu^2+^-AA mixtures. The first column (i) represents the responses for Cu^2+^ in each solution while the second column (ii) shows responses for 5-HT, DA and AA respectively. Each experiment was conducted on at least four electrodes with three replicates (minimum of 12 replicates), and the average highest oxidation/reduction current for each analyte was plotted with ± standard error of the mean.

**Fig. 5:**
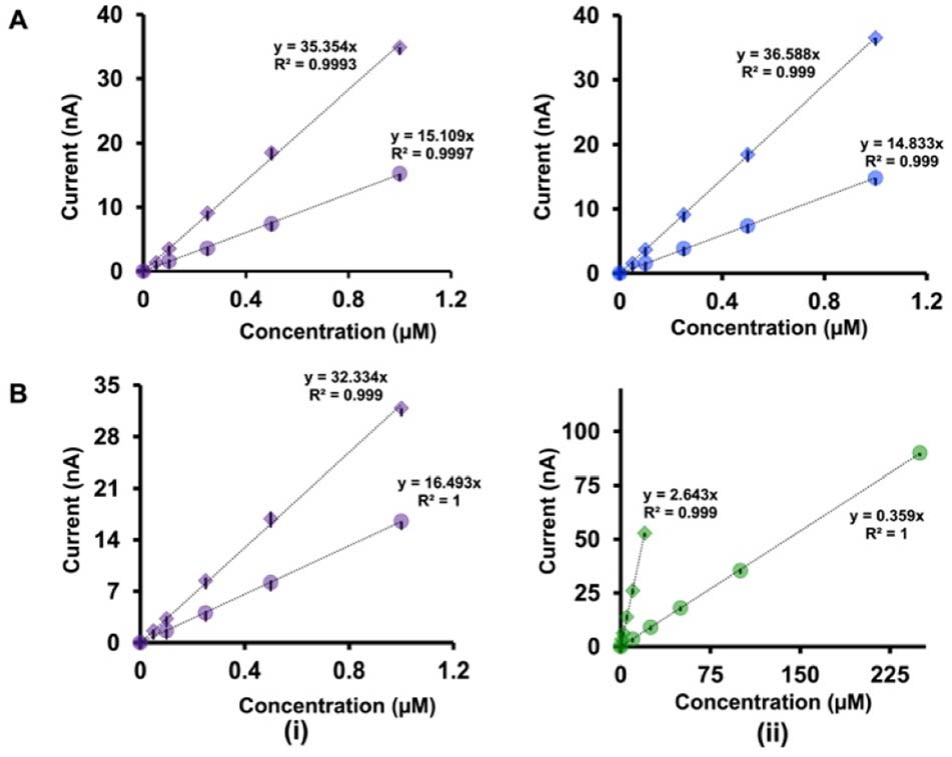
Comparison of the linear ranges of calibration curves obtained with double-bore CFMs by applying two waveforms simultaneously (diamonds) and with single-bore CFMs by applying one waveform at a time (circles) for (A) 5-HT-DA, and (B) 5-HT-AA. The first column (i) represents the responses for 5-HT in each solution while the second column (ii) shows responses for DA and AA respectively. Each experiment was conducted on at least four electrodes with three replicates (minimum of 12 replicates), and the average highest oxidation/reduction current for each analyte was plotted with ± standard error of the mean.

The analysis of the calibration curves obtained with our double-bore CFMs and single-bore CFMs yields important information about the performance of our electrodes. Fig. 4 shows only the linear ranges for Cu^2+^-5-HT (Fig. 4A), Cu^2+^-DA (Fig. 4B) and Cu^2+^-AA (Fig. 4C) obtained with double-bore and single-bore CFMs while Fig. 5 shows those obtained for 5-HT-DA (Fig. 5A) and 5-HT-AA (Fig. 5B) mixtures. Similarly, Fig. S6 and S7† depict full calibration curves.

Moreover, Tables 1(A) and (B) summarize the LOD, linear ranges, and sensitivities for each solution mixture with double-bore CFMs and single-bore CFMs. Except for the Cu^2+^-DA solution, the analytical parameters for all other analyte mixtures were significantly improved when those were simultaneously detected with double-bore CFMs compared to single-bore CFMs. For example, as seen in Table 1(B), the LOD of Cu^2+^ and 5-HT, with single-bore CFMs, are 0.5 μM and 0.05 μM, respectively, and it was improved by two orders of magnitude (0.25 μM and 0.025 μM for Cu^2+^ and 5-HT respectively) when those two were detected simultaneously with double-bore CFMs. Among all the analyte mixtures tested, the lowest LOD (0.025 μM) was found with 5-HT when co-detecting with Cu^2+^. The sensitivity was doubled for both analytes upon co-detection with double-bore CFMs. Interestingly, the most remarkable improvement was seen in the 5-HT-AA solution mixture. The LOD of AA was spiked to 1 μM with double-bore compared to 10 μM with single-bore CFMs. Similarly, the sensitivity of AA in the same mixture was increased almost by ten orders of magnitudes upon simultaneous detection with 5-HT compared to detecting each analyte one at a time (Fig. 5B(ii)). At this point, we hypothesize that this increase in the analytical performance of double-bore CFMs is primarily due to the presence of a secondary electric field on the adjacent electrode in close proximity (∼nm) and its ability to capture the oxidative or the reductive product of one analyte and cycle back to its initial state, thus, preventing or minimizing the diffusion of the analyte away from the electrode surface.

As mentioned above, there's no significant improvement in our double-bore CFM for the simultaneous detection of Cu^2+^-DA, and this could presumably be due to a stable complex formed between Cu^2+^-DA in the presence of an electric field, thus, not cycling either of the analytes back to their original state. Moreover, mussel-inspired chemistry has found that DA is able to self-polymerize into polydopamine (PDA), and PDA can act as a bio glue for heavy metals such as Cu^2+^.^44,45^ In particular, PDA has been previously utilized as a biocompatible surface modification on CFMs to enhance the detection of Cu^2+^ using FSCV.^36^

## Conclusions

The inadequate understanding of the effects of co-transmission and environmental factors, such as heavy metals, on neurodegenerative diseases highlights the importance of developing a sensor capable of performing simultaneous, multi-analyte detection in real-time. Although FSCV has been well-established for detecting a single neurotransmitter, studies into multi-analyte detection are minimal. Moreover, the fabrication protocols for electrodes capable of performing such multi-analysis are lengthy and meticulous and require significant changes to detection parameters. Furthermore, these studies do not include detecting other environmental factors, such as toxic metals, which play a crucial role in neuronal cell death. In this study, we fabricated a simple double-bore CFM capable of the simultaneous detection of both neurotransmitters and toxic metals using FSCV. We made our electrode manually *via* a simple, cheap procedure, and the gap between two fibers was identified *via* SEM images. We obtained oscilloscope images of our double-bore sensor and tested our sensor's ability to withstand two waveforms applied simultaneously. After confirming the stability of our sensor, we performed FSCV measurements with different combinations of our analytes: Cu^2+^-DA, Cu^2+^-5-HT, Cu^2+^-AA, 5-HT-DA, and 5-HT-AA by applying analyte-specific waveforms and constructed calibration curves. Additionally, we performed control tests with single-bore CFMs in those solution mixtures by applying one waveform at a time. Our double-bore CFMs resulted in enhanced analytical parameters in all analyte mixtures except for Cu^2+^-DA solution. There's no significant difference seen in the analytical performance between single-bore CFMs and double-bore CFMs in Cu^2+^-DA solution. We attribute the enhanced detection parameters of our double-bore CFMs to the cycling of the oxidative/reductive products to their original state by the additional waveform on the adjacent electrode, thereby minimizing their diffusion from the electrode surface. We also hypothesize that Cu^2+^ and DA make a stable complex; thus, there is no cycling to the original states, resulting in the same sensitivity. To the best of our knowledge, this is the first time reporting a double-bore CFM capable of detecting two analytes using analyte-specific waveforms at fast scan rates with no electrical interference from the adjacent electrode. Although the exact mechanism of our sensor is yet to be explored, data from this study showcases the strength of our double-bore CFM to be used for future *in vivo* studies with improved LOD, linear ranges, and sensitivities for detecting two analytes simultaneously in real-time *via* FSCV, thus, allowing us to obtain missing information about the complex etiology of neurodegenerative diseases.

## Conflicts of interest

There are no conflicts to declare.

## Supplementary Material

RA-013-D3RA06218J-s001
