# Supplementary material for: Simultaneous detection of neurotransmitters and Cu2+ using double-bore carbon fiber microelectrodes via fast-scan cyclic voltammetry
Source: RSC Adv. 2023 Nov 20;13(48):33844–51. doi: 10.1039/d3ra06218j (PMC10658548; doi:10.1039/d3ra06218j)
Supplement: RA-013-D3RA06218J-s001 [file RA-013-D3RA06218J-s001.pdf]

Electronic Supplementary Material (ESI) for RSC Advances.
This journal is © The Royal Society of Chemistry 2023

Supporting Information

Simultaneous Detection of Neurotransmitters and Cu^{2+} using Double-bore Carbon Fiber Microelectrodes Via Fast - Scan Cyclic Voltammetry

Noel Manring[a], Miriam Strini[a], Jessica L. Smeltz[a], Pavithra Pathirathna[a]*

[a]Department of Chemistry and Chemical Engineering, Florida Institute of Technology, Melbourne, FL

*Email: ppathirathna@fit.edu

Contents:

1. Optical microscopic image of double-bore CFM
2. Calibration curves for co-detection of Cu^{2+}-5-HT, Cu^{2+}-DA, and Cu^{2+}-AA with double-bore CFMs
3. Calibration curves for co-detection of 5-HT-DA and 5-HT-AA with double-bore CFMs
4. Color plots for co-detection of Cu^{2+}-5-HT, Cu^{2+}-DA, and Cu^{2+}-AA with double-bore CFMs
5. Color plots for co-detection of 5-HT-DA and 5-HT-AA with double-bore CFMs
6. Calibration curves for analyte mixtures of Cu^{2+}-5-HT, Cu^{2+}-DA, and Cu^{2+}-AA with single-bore CFMs
7. Calibration curves for single-bore analyte mixtures of 5-HT-DA and 5-HT-AA with single-bore CFMs

Opitical microscopic image of double-bore CFM - After fabricating the double-bore CFMs, we analyzed it under the optical microscope (Unitron Examet-5 series, Commack, NY, USA). As seen in Figure S1, there is an identifiable gap between the two carbon fibers.

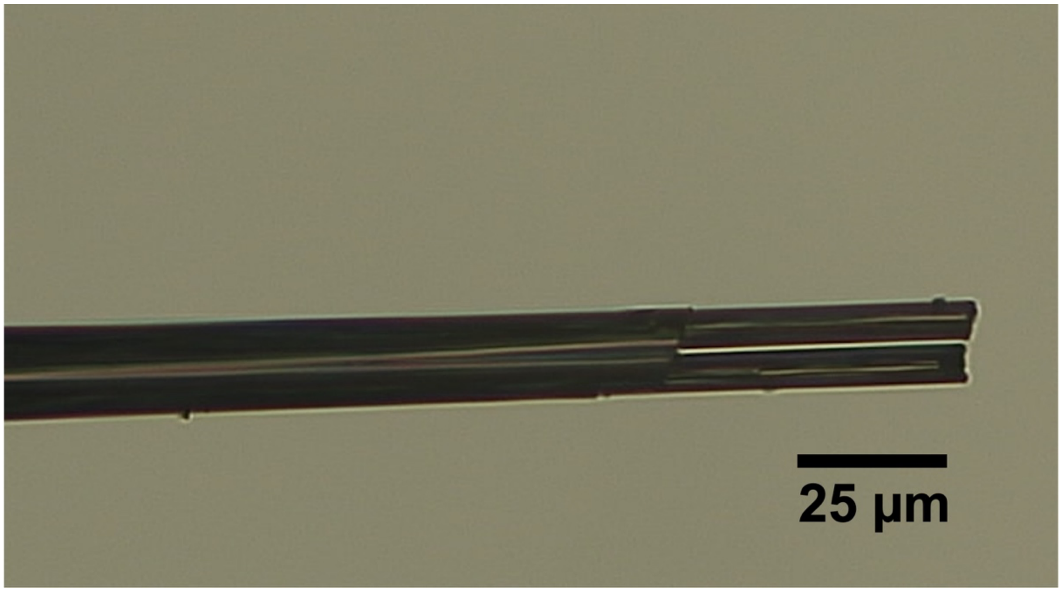

Figure S1: Optical microscope image of double-bore CFM.

Calibration curves for co-detection of Cu^{2+}-5-HT, Cu^{2+}-DA, and Cu^{2+}-AA with double-bore CFMs - After confirming the stability of our double-bore CFMs, we performed simultaneous FSCV measurements of Cu^{2+}-5-HT, Cu^{2+}-DA, and Cu^{2+}-AA in tris buffer.

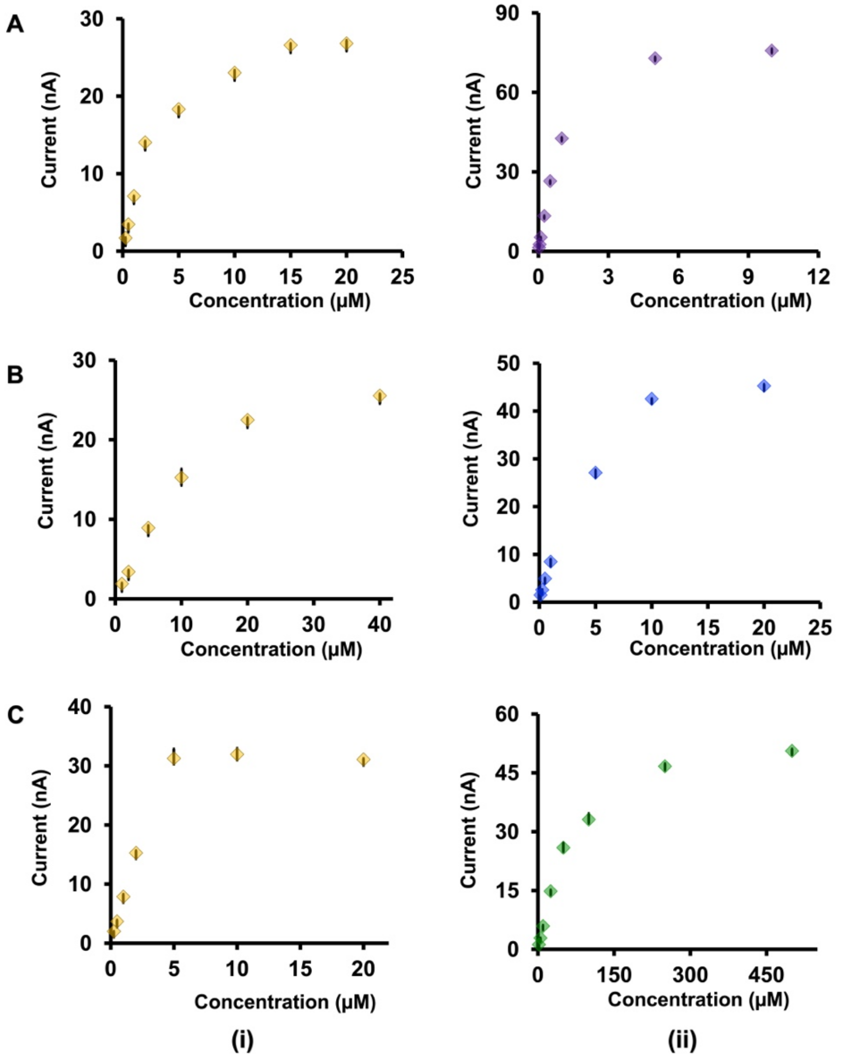

Figure S2: Full calibration curves obtained with double-bore CFMs by applying two waveforms simultaneously with double-bore CFMs for (A) Cu^{2+}-5-HT (B) Cu^{2+}-DA, (C) and Cu^{2+}-AA mixtures. The first column (i) represents the responses for Cu^{2+} in each solution while the second column (ii) shows responses for 5-HT, DA and AA respectively. Each experiment was conducted on at least four electrodes with three replicates (minimum of 12 replicates), and the average highest oxidation/reduction current for each analyte was plotted with ± standard error of the mean.

Calibration curves for co-detection of 5-HT-DA and 5-HT-AA with double-bore CFMs - We also performed simultaneous FSCV measurements for 5-HT-DA and 5-HT-AA in tris buffer. Figure S3 shows the complete calibration curves for those two solutions.

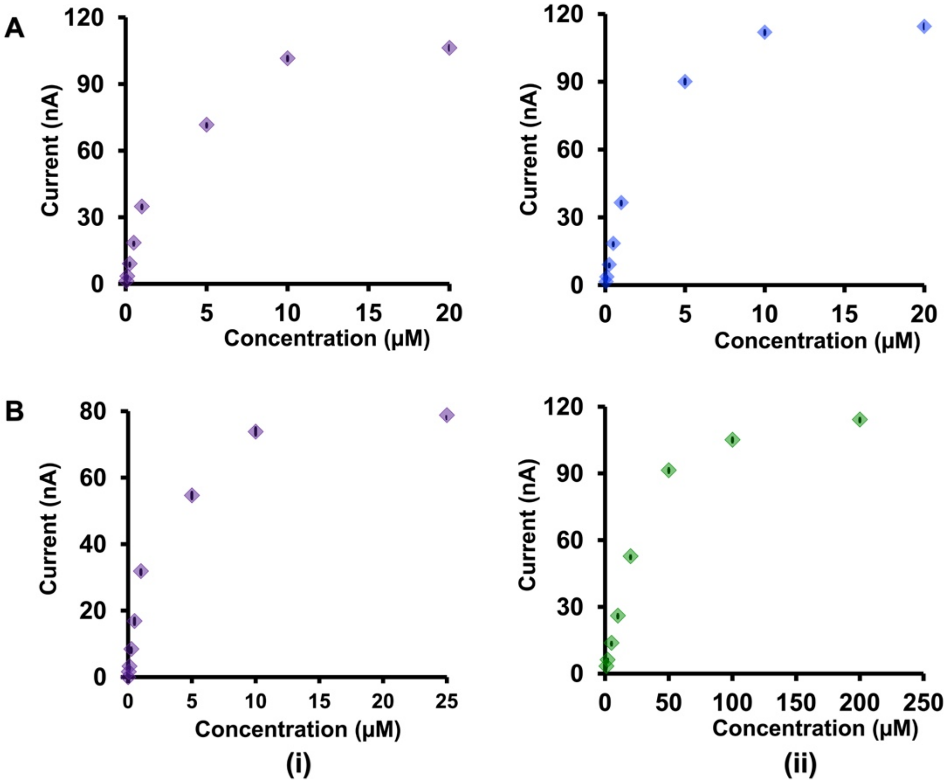

Figure S3: Full calibration curves obtained with double-bore CFMs by applying two waveforms simultaneously for (A) 5-HT-DA, and (B) 5-HT-AA. The first column (i) represents the responses for 5-HT in each solution while the second column (ii) shows responses for DA and AA respectively. Each experiment was conducted on at least four electrodes with three replicates (minimum of 12 replicates), and the average highest oxidation/reduction current for each analyte was plotted with ± standard error of the mean.

Color plots for co-detection of Cu^{2+}-5-HT, Cu^{2+}-DA, and Cu^{2+}-AA with double-bore CFMs - Each FSCV experiment was conducted for 20 seconds, and the following are the representative color plots obtained for (A) Cu^{2+}-5-HT, (B) Cu^{2+}-DA and (C) Cu^{2+}-AA mixtures upon detecting both analytes simultaneously with double-bore CFMs.

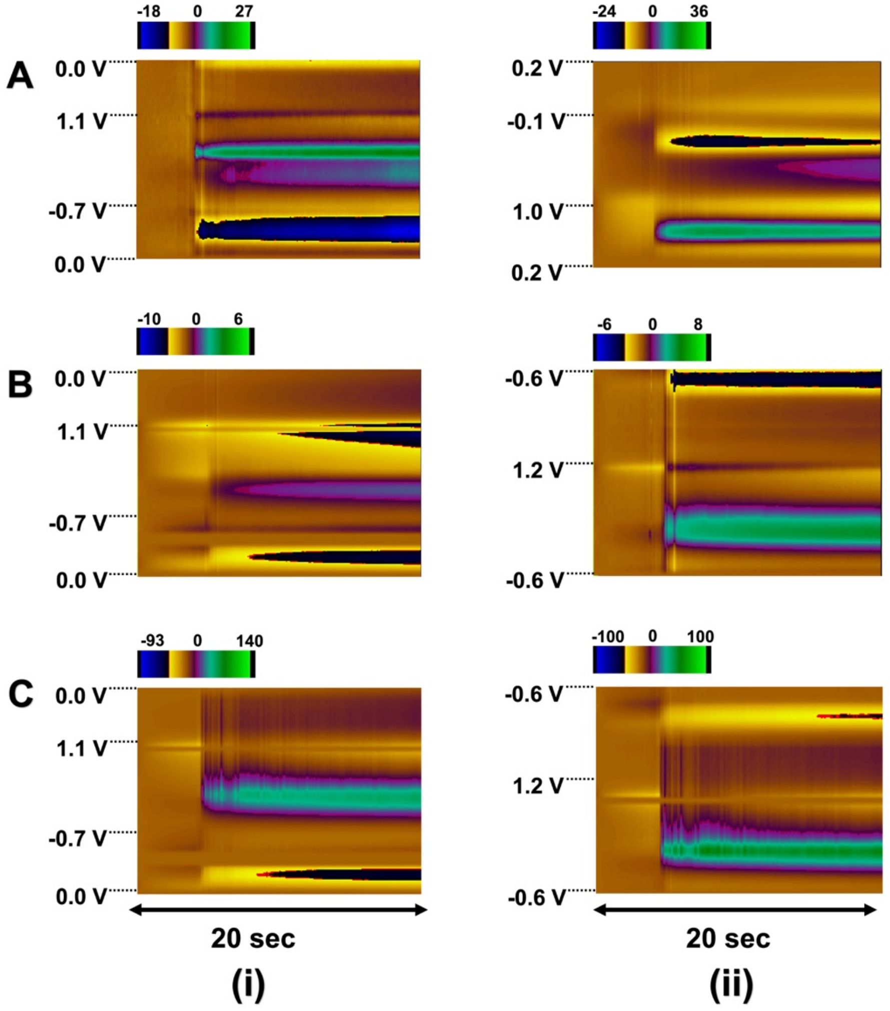

Figure S4: Representative Color plots for the double-bore co-detection of Cu^{2+}-5-HT, Cu^{2+}-DA and Cu^{2+}-AA analyte mixtures. The first column (i) illustrates Cu^{2+} color plots and the second column (ii) represent color plots for 5-HT, DA and AA.

Color plots for co-detection of 5-HT-DA and 5-HT-AA with double-bore CFMs - We also collected color plots for the co-detection of 5-HT-DA and 5-HT-AA with our double-bore CFMs.

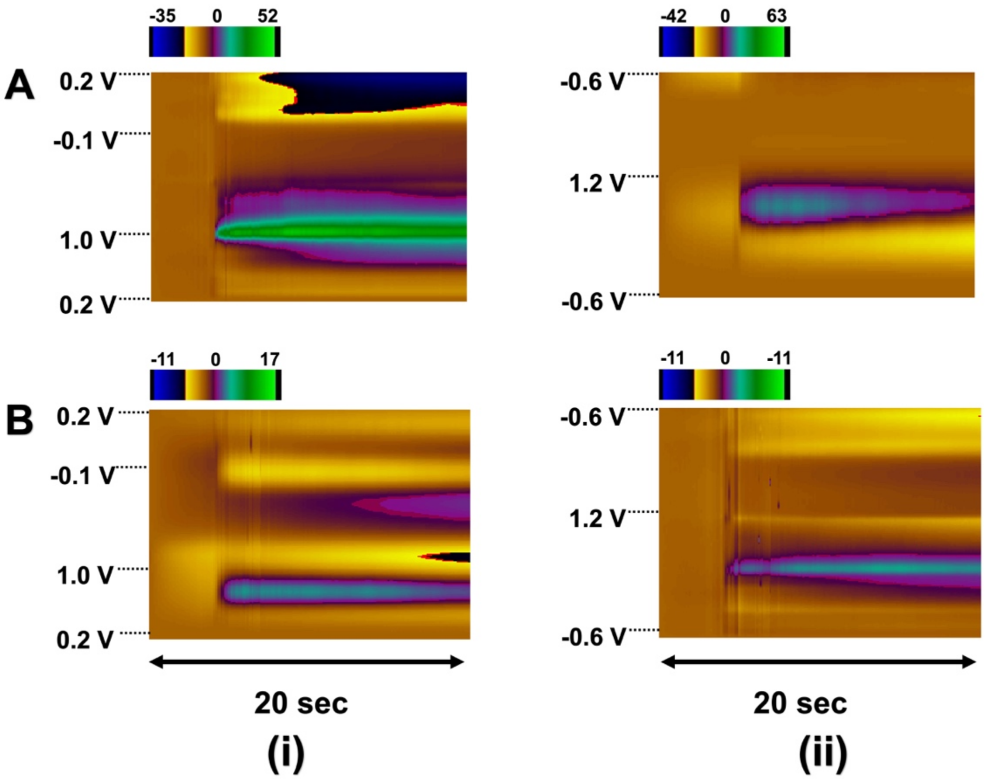

Figure S5: Representative color plots for the double-bore co-detection of 5-HT-DA, and 5-HT- AA analyte mixtures. The first column (i) illustrates 5-HT color plots and the second column (ii) represent color plots for DA and AA.

Calibration curves for analyte mixtures of Cu^{2+}-5-HT, Cu^{2+}-DA, and Cu^{2+}-AA with single-bore CFMs- We constructed calibration curves obtained for the same analyte mixtures with single-bore CFMs by applying one waveform at a time to compare the analytical performance of our double-bore CFMs.

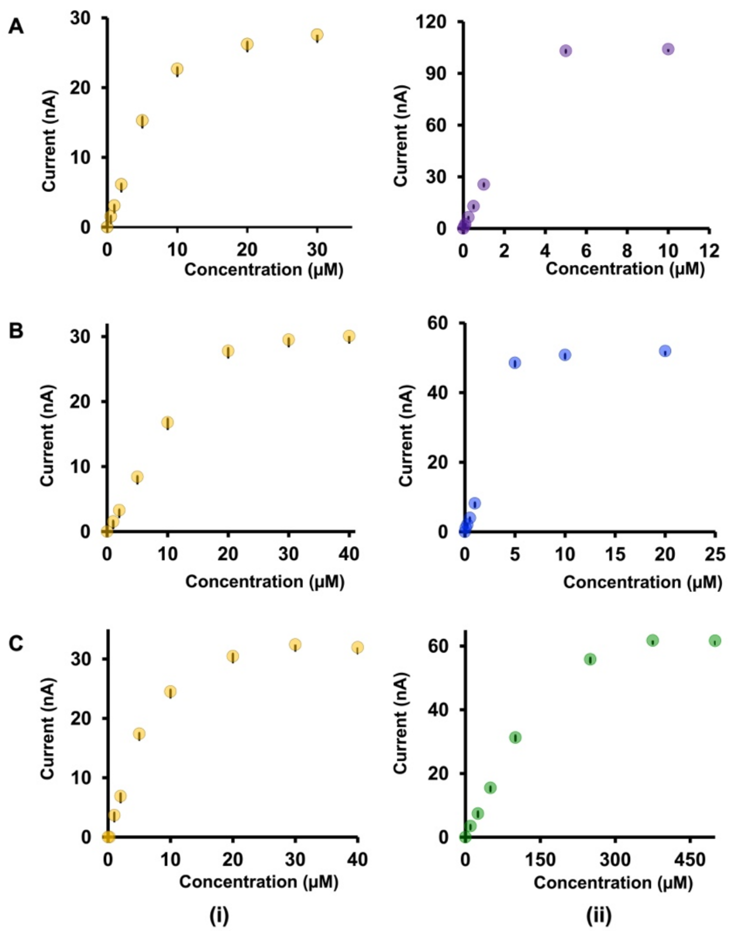

Figure S6: Full calibration curves obtained with single-bore CFMs by applying one waveform at a time for (A) Cu^{2+}-5-HT, (B) Cu^{2+}-DA and (C) Cu^{2+}-AA. The first column (i) represents the responses for Cu^{2+} in each solution while the second column (ii) shows responses for 5-HT, DA and AA respectively. Each experiment was conducted on at least four electrodes with three replicates (minimum of 12 replicates), and the average highest oxidation/reduction current for each analyte was plotted with ± standard error of the mean.

Calibration curves for analyte mixtures of 5-HT-DA and 5-HT-AA with single-bore CFMs- We also analyzed 5-HT-DA and 5-HT-AA mixtures with single-bore CFMs. Figure S7 shows the complete calibration curves for these analytes in tris buffer.

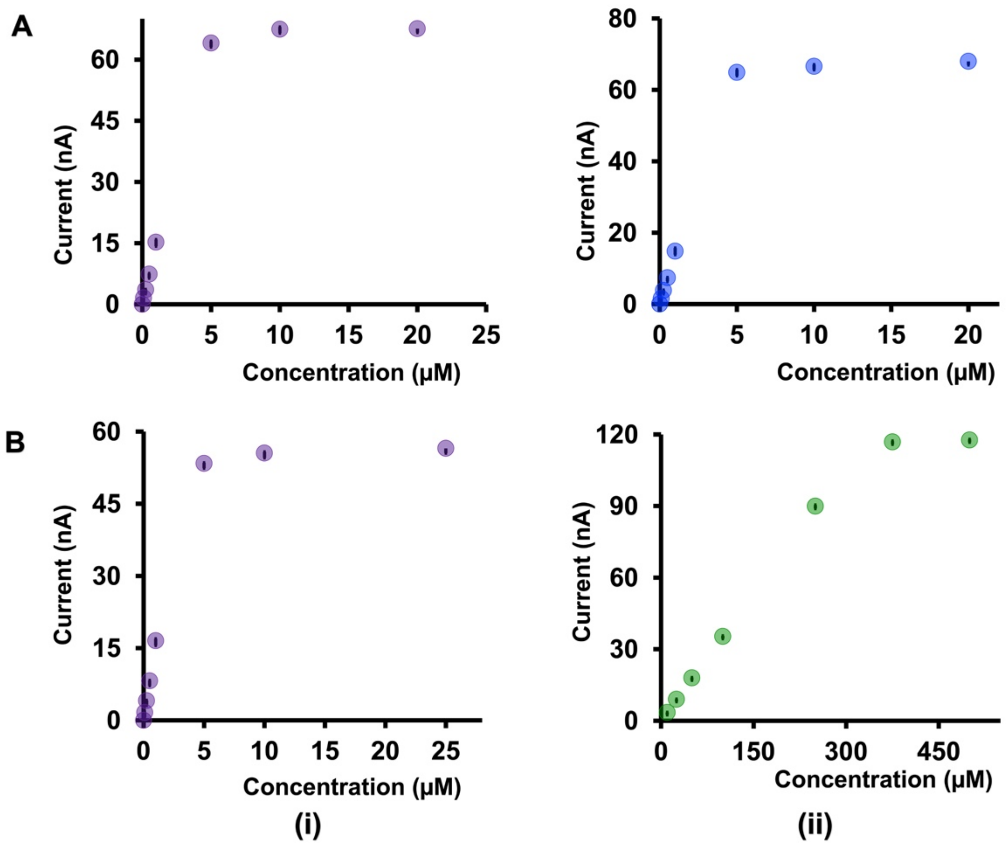

Figure S7: Full calibration curves obtained with single-bore CFMs by applying one waveform at a time for (A) 5-HT-DA, and (B) 5-HT-AA. The first column (i) represents the responses for 5-HT in each solution while the second column (ii) shows responses for DA and AA respectively. Each experiment was conducted on at least four electrodes with three replicates (minimum of 12 replicates), and the average highest oxidation/reduction current for each analyte was plotted with ± standard error of the mean.
